# Driving Performance Under Treatment of Most Frequently Prescribed Drugs for Mental Disorders: A Systematic Review of Patient Studies

**DOI:** 10.1093/ijnp/pyab031

**Published:** 2021-05-26

**Authors:** Alexander Brunnauer, Florian Herpich, Peter Zwanzger, Gerd Laux

**Affiliations:** 1 kbo-Inn-Salzach-Klinikum, Clinical Center for Psychiatry, Psychotherapy, Psychosomatic Medicine, Geriatrics and Neurology, Wasserburg/Inn, Germany; 2 Department of Psychiatry and Psychotherapy, Ludwig-Maximilians University, Munich, Germany; 3 Institute of Psychological Medicine (IPM) Soyen, Germany

**Keywords:** Antidepressants, antipsychotics, benzodiazepines, driving performance, mood-stabilizers

## Abstract

**Background:**

Mobility is important for daily life functioning, with particular challenges regarding road safety under pharmacological treatment in patients with a psychiatric disease.

**Methods:**

According to PRISMA guidelines, a systematic literature search on PubMed database (January 1970 to December 2020) was performed. Primary endpoints were driving performance in on-road tests, driving simulator performance, or psychomotor and visual perception functions assessed to estimate fitness to drive according to legal regulations in patient studies.

**Results:**

Forty studies were identified (1533 patients, 38% female, median age 45 years), of which more than 60% were cross-sectional and open-label trials. Under steady-state medication, 31% (range 27%–42.5%) of schizophrenic or schizoaffective patients under antipsychotics and 18% (range 16%–20%) of unipolar and bipolar patients under antidepressants showed severe impairment in skills relevant for driving. Data point to an advantage of second-generation antipsychotics compared with first-generation antipsychotics as well as modern antidepressants over tricyclic antidepressants with respect to driving. Most patients significantly improved or stabilized in driving skills within 2–4 weeks of treatment with non-sedative or sedative antidepressants. Diazepam significantly worsened driving the first 3 weeks after treatment initiation, whereas medazepam (low dose), temazepam, and zolpidem did not impair driving. In long-term users of sedating antidepressants or benzodiazepines, impairments in on-road tests were not evident.

**Conclusion:**

The available evidence suggests that psychopharmacologic medicines improve or at least stabilize driving performance of patients under long-term treatment when given on clinical considerations. To enhance treatment compliance, existing classification systems of medicinal drugs concerning impact on driving performance should also incorporate information about effects of long-term-treatment.

## Introduction

Modern societies demand a high grade of mobility, and there is evidence that driving cessation, for example, in cases of aging or chronic illness, affects social and economic well-being with impacts on health functioning (Edwards et al., 2010). Approximately 67% of patients with a psychiatric disease have a drivers’ license; 77% of these patients report driving regularly with their cars and most of them (88%) use prescribed medication. Closer inspection of data also indicates that driving restrictions largely affect the social functioning of these patients (Brunnauer et al., 2016). Thus, road safety under pharmacological treatment is of great relevance for patients with a psychiatric disease and is therefore frequently discussed in clinical practice.

Besides their major mode of action, medicinal drugs can impair behavior most frequently because of sedating CNS adverse effects such as drowsiness or inability to concentrate. Behavioral correlates of drug-induced impairments may be a disruption of neuropsychological processes controlling behavior. Evidence supporting the concept of behavioral toxicity (e.g., DiMascio and Shader, 1968; Hindmarch I, 1994) comes from epidemiological data. Albeit causal relationships can hardly be drawn, there is considerable evidence that the use of psychoactive drugs is associated with an increased risk of traffic injuries, with a particular concern regarding benzodiazepines and tricyclic antidepressants (e.g., Ray et al., 1992; Leveille et al., 1994; Mura et al., 2003; Barbone et al., 1998; Bramness et al., 2008; Dassanayake et al., 2011; Elvik, 2013). Especially elderly users of sedating antidepressants have a more than twofold increased risk of being involved in road traffic accidents (Ray et al., 1992; Leveille et al., 1994; Dassanayake et al., 2011). Benzodiazepine use and the association with traffic accidents seems to be more frequent in younger drivers, and risk markedly increased by co-ingestion of alcohol (Dassanayake et al., 2011).

Empirical evidence from experimental studies also indicates that behavioral toxicity, particularly with sedating CNS effects in the acute phase of treatment, is of major concern with respect to driving performance (Ramaekers, 2003; Rapoport and Baniña, 2007; Berghaus et al., 2010; Strand et al., 2016; Brunnauer and Laux, 2017). However, cognitive dysfunction per se is also a core feature of many psychiatric disorders that is prominent in untreated patients and thus not necessarily linked to medication effects (Millan et al., 2012; Rock et al., 2014). Besides, there is evidence that long-term treatment with, for example, antidepressants or antipsychotics may help to improve psychomotor and cognitive dysfunction in patients with a psychiatric disease (Woodward et al., 2005; Keefe et al., 2007, 2014; Baune and Renger, 2014; Nielsen et al., 2015; Rosenblat et al., 2015; Prado et al., 2018).

Taken together stabilizing effects of pharmacological treatment have to be weighed against possible detrimental cognitive, vegetative-somatic, and psychomotor effects when validating drugs with respect to driving performance. Studies on this topic usually have investigated acute effects of medicines in healthy individuals that may be quite different from issues of long-term treatment of patients. Thus, the crucial question of which pharmacological treatment patients benefit most with respect to driving performance can only be validly answered by patient studies.

This systematic review gives an update of available evidence of patient studies on treatment effects on driving ability in psychiatric patients of the most frequently prescribed psychotropic drugs.

## Methods

A systematic literature search in the PubMed database (January 1970 to December 2020) was performed using a combination of subject headings and keyword terms: “driving performance,” “driving ability,” “driving skills,” “fitness to drive,” “traffic safety,” “on the road driving test,” “driving simulation” AND “antidepressants,” “antipsychotics,” “benzodiazepines,” “Z-drugs,” “MAO-inhibitors,” and “mood-stabilizers.” Furthermore, combinations of additional search terms were included to screen for further articles (search terms are listed in supplementary Materials). The titles and, if relevant, the abstracts of each citation were screened manually, and the full texts of relevant citations were then analyzed in detail. Besides, reference lists were manually searched for relevant studies. All identified articles were screened and, if necessary, discussed by 2 independent reviewers (Drs Brunnauer and Herpich).

## Types of Outcome Measures

We integrated evidence of various research methodologies that focused on driving performance in psychiatric patients. Articles were selected if they (1) examined driving performance in an on-road test in real traffic or in a simulated on-road test, that is, on a closed circuit; (2) used a driving simulator with at least medium complexity, that is, a fixed hardware consisting of a steering wheel, brake pedals and simulated road environment; or (3) investigated psychomotor and visual perception functions to investigate driving skills according to legal regulations.

**Figure 1.**
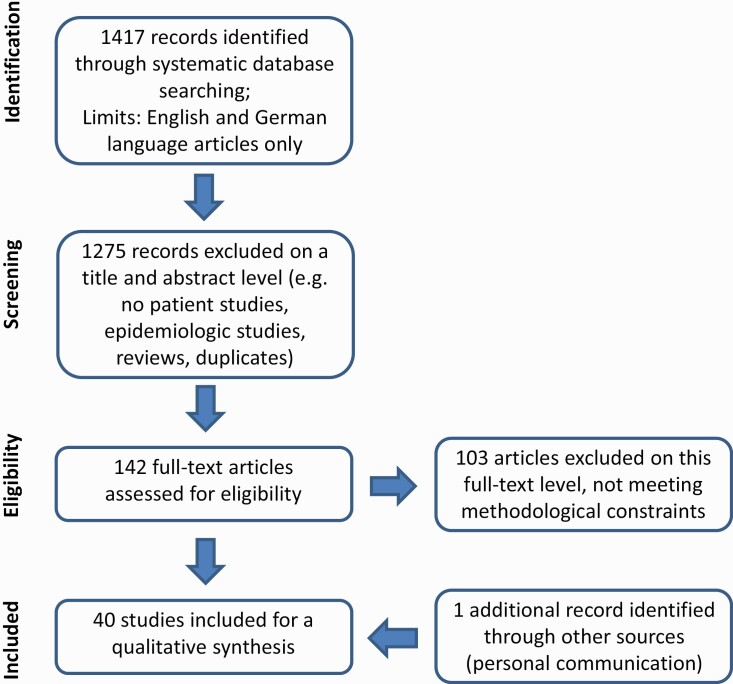
Summary of the search strategy according to PRISMA guidelines

Articles in English or German language, in which study design, sample, and methods used had been described adequately, were considered for this review. The search results and study selection process are summarized in the following PRISMA flowchart (see Figure 1).

## Results

### Study Selection

A total of 1417 hits were identified using the search terms mentioned above. The screening of titles, abstracts, and full-text articles (in a final step the analysis) of this literature search revealed 39 articles assessing driving ability with methodologies described above. Reference lists of the included studies and related reviews did not lead to an inclusion of further studies. Personal communication led to an inclusion of 1 further study.

### Study Description

Forty studies were identified according to selection criteria. Patients were diagnosed according to ICD-10, DSM-III-R, or DSM-IV criteria. Additionally, a smaller group of patients met the criteria by structured interviews and rating scales. Studies consisted of 25 cross-sectional or open-label trials, 3 randomized controlled studies, and 12 investigations in a (randomized) double-blind design. The distribution of patients investigated in an inpatient setting (40%) was comparable with those investigated in an outpatient setting (42%); treatment setting was not specified in 18% of studies under review.

#### Antipsychotics

Twelve studies were included in this review with schizophrenic, schizoaffective, manic depression, and borderline patients. The median sample size was 31 (range 22–120) with a total of 536 patients (37% females). The median age was 33 years (range 18–62 years). All investigations were designed as comparative clinical trials (cross-sectional and open label).

#### Antidepressants

The effects of antidepressants on driving performance were assessed in 14 clinical studies comprising 587 patients with primarily depressive disorders (44% female) with a median age of 45 years (range 18–78 years). Eight of these trials investigated driving performance within an open-label design, and in 6 trials patients were randomly assigned to treatment groups (4 under double-blind conditions).

#### Mood-Stabilizers

Three publications were identified investigating driving performance in bipolar and unipolar patients and a group of patients with Meniere’s disease. Sixty patients were investigated (48% female) with a median age of 45 years (range 18–63 years). Studies were designed as cross-sectional or open-label trials.

#### Benzodiazepines and Z-Drugs

Eleven studies investigated the effects of benzodiazepines or Z-drugs on driving performance in patients with an anxiety disorder or patients with insomnia. The median sample size was 18 (range 12–44) with a total of 407 patients (22% females). The median age was 40 years (range 18–50 years) in the groups treated with tranquilizers and 55 years (range 25–75 years ) in studies investigating hypnotics. Most studies were designed as (randomized) double-blind cross-over trials. An overview of studies included is provided in Table 1.

**Table 1.** Summary of patient studies included using (S)ORT or DS or investigating PP according to legal regulations

Authors (year)	Investigational drugs	Dose	Status	Age (y)	Design	Test	Outcome^*a*^
Antipsychotics							
Comparative clinical studies							
Wylie et al. (1993)	Flupenthixol (dec.) Fluphenazine (dec.) + procyclidine comedication	n. spec.	Schizophrenic patients (n = 22)	21–62	Other	DS	Long-term treatment (n. spec.) = significant lower performance of patients compared with healthy controls No difference between treatment groups
Soyka et al. (2001)	Haloperidol Risperidone (8 patients received biperiden comedication	5–30 mg 2–6 mg	Schizophrenic patients (n = 26)	21–46	Other	PP	Long-term treatment (n. spec.) = better performance of patients treated with risperidone
Brunnauer et al. (2004)	FGAs SGAs Flupenthixol Clozapine (7 patients received biperiden comedication	Mean dose (CPÄ) 464.7 mg 535.2 mg 408.0 mg 528.6 mg	Schizophrenic and schizoaffective patients (n = 120)	18–59	Other	PP	Long-term treatment (n. spec.) = 27% severely impaired Better performance of patients treated with SGAs (respectively clozapine)
Brunnauer et al. (2005)	Haloperidol Flupenthixol Risperidone (4 patients received biperiden comedication	Mean dose 6.3 mg 5.5 mg 4.1 mg	Schizophrenic and schizoaffective patients (n = 47)	21–60	Other	PP DS	Long-term treatment (n. spec.) = 32% severely impaired Better performance of patients treated with risperidone Long-term treatment (n. spec.) = advantage for patients treated with risperidone in risk simulation
Soyka et al. (2005)	Haloperidol Risperidone (11 patients received biperiden comedication	5–30 mg 4–8 mg	Schizophrenic and schizoaffective patients (n = 40)	33.1 (mean age) 32.8 (mean age)	Other	PP	Long-term treatment (n. spec.) = 42.5% of patients showed very low performance Better performance of patients treated with risperodone
Brunnauer et al. (2009)	Amisulpride Quetiapine Flupenthixol Haloperidol	200–800 mg 300–800 mg 3–10 mg 2–15 mg	Schizophrenic patients (n = 80)	18–60	Other	PP DS	Long-term treatment (n. spec.) = 27.5% severely impaired Better performance of patients treated with amisulpride or quetiapine, especially in vigilance and concentration Long-term treatment (n. spec.) = advantage for patients treated with amisulpride or quetiapine in risk simulation
Brunnauer and Laux (2012)	Sertindole Risperidone Quetiapine	8–20 mg 2–8 mg 300–500 mg	Schizophrenic patients (n = 30)	21–52	Other	PP	Long-term treatment (n. spec.) = 27% severely impaired No significant differences between treatment groups
Treatment combinations							
Hobi et al. (1981)	FGAs + comedication	n. spec.	Schizohrenic patients, manic depression, borderline (n = 30)	20–50	Other	DS	Subchronic and long-term treatment (n. spec.) = patients did not approach level of healthy controls
Grübel-Mathyl (1987)	Combinations of FGAs	26 on depot neuroleptics, 7 on oral medication	Schizophrenic and schizoaffective patients (n = 33)	20–44	Other	PP	Long-term treatment (n. spec.) = significant lower performance of patients compared with healthy controls
Grabe et al. (1999)	Haloperidol Perazine Chlorprothixene Flupenthixol Clozapine Amisulpride + comedication	Mean dose 9 mg 313 mg 50 mg 13 mg 290 mg 350 mg	Schizophrenic patients (n = 28)	32,3 (mean age)	Other	PP	Long-term treatment (n. spec.) = 32% severely impaired Significantly better test performance of patients under polydrug treatment with clozapine in reactivity and stress tolerance compared with FGAs
Kagerer et al. (2003)	Haloperidol Amisulpride Clozapine Quetiapine Risperidone Olanzapine Ziprasidone + comedication	4–30 mg 400–1000 mg 125–450 mg 200–1200 mg 3–10 mg 20–25 mg 80 mg	Schizophrenic and schizoaffective patients (n = 49)	19–49	Other	PP	Long-term treatment (n. spec.) = 54% showed very low performance with advantage for patients treated with SGAs
Fuermaier et al. (2019)	Antipsychotics + comedication	Mean dose 8.7 mg (haloperidol equivalent dose)	Schizophrenic patients (n = 31)	29,9 (mean age)	Other	DS	Long-term treatment (n. spec.) = patients drove slower than healthy controls; no indication of deviant driving
Antidepressants							
Veldhuijzen et al. (2006)	Amitriptyline	25 mg	Chronic neuropathic pain patients (n = 7)	42–58	RDBC	ORT	Acute treatment (n) = impairment Subchronic treatment (n) = no impairment
Brunnauer et al. (2015)	Agomelatine	25–50 mg	Depressive patients (n = 20)	50,4 (mean age)	RCT	PP ORT	Subchronic treatment (n. spec.) = improvement Long-term treatment (n. spec.) = stabilization Depressed patients inferior to healthy controls Prior discharge depressed patients not significantly inferior to healthy controls
Brunnauer et al. (2008)	Mirtazapine	30–60 mg	Depressive patients (n = 20)	25–67	RCT	PP DS	Subchronic treatment (n. spec.) = improvement Depressed patients inferior to healthy controls Depressed patients not inferior to healthy controls
Shen et al. (2009)	Mirtazapine	30 mg	Depressive patients (n = 14)	29–67	RCT	DS	Acute treatment (n) = improvement Long-term treatment (n) = improvement
Brunnauer et al. (2008)	Reboxetine	2–8 mg	Depressive patients (n = 20)	38–56	RCT	PP DS	Subchronic treatment (n. spec.) = improvement Depressed patients not inferior to healthy controls
Roth et al. (2011)	Trazodone	50 mg	Primary insomniacs (n = 16)	18–65	RDBC	DS	Acute treatment (n) = no impairment (high drop-out rate)
Brunnauer et al. (2015)	Venlafaxine	150–300 mg	Depressive patients (n = 20)	51,1 (mean age)	RCT	PP ORT	Subchronic treatment (n. spec.) = improvement Long-term treatment (n. spec.) = stabilization Depressed patients inferior to healthy controls Prior discharge depressed patients not significantly inferior to healthy controls
Comparative clinical studies							
Brunnauer et al. (2006)	Amitriptyline Doxepin Marprotiline Trimipramine Citalopram Paroxetine Mirtazapine Venlafaxine	60–180 mg 75–225 mg 100–150 mg 100–150 mg 20–40 mg 20–50 mg 20–60 mg 100–150 mg	Depressive and bipolar patients (n = 100)	20–78	Other	PP	Long-term treatment (n. spec.) = 16% of patients showed severe impairment TCAs > SSRIs and mirtazapine TCAs vs venlafaxine = no difference Mirtazapine < SSRIs
Wingen et al. (2006)	Citalopram Paroxetine Sertraline Venlafaxine	20–40 mg 20–40 mg 50–100 mg 75–300 mg	Depressive patients (n = 24)	21–63	Other	ORT	Long-term treatment (n. spec.) = inferior to healthy controls No differences between treatment groups
van der Sluiszen et al. (2017b)	Citalopram Paroxetine Sertraline Venlafaxine	20–40 mg 20–40 mg 50–100 mg 75–300 mg	Depressive patients (n = 65)	21–63	Other	ORT	Long-term treatment (n. spec.) = better performance than untreated patients Untreated and treated patients inferior to healthy controls
Treatment combinations							
Hobi et al. (1981)	Tri– and tetracyclic antidepressants + comedication	n. spec.	Depressive and bipolar patients (n = 31)	20–50	Other	DS	Subchronic and long-term treatment (n. spec.) = approached level of healthy controls
Ramaekers et al. (1997)	Fluoxetine + BZD	20–40 mg	Depressive patients (n = 17)	18–32	RDB	ORT	Long-term treatment (n. spec.) = impairment in patients with competitive benzodiazepine comedication at wk 3
Ramaekers et al. (1997)	Moclobemide + BZD	300–600 mg	Depressive patients (n = 22)	27–55	RDB	ORT	Long-term treatment (n. spec.) = impairment in patients with competitive benzodiazepine comedi–cation
Grabe et al. (1998)	Amitriptyline Desipramine Trimipramine Imipramine Clomipramine Maprotiline Mianserin Trazodone Fluoxetine Fluvoxamine Paroxetine Moclobemid Tranylcypromine + comedication	125–250 mg 100 mg 100 mg 150 mg 150–300 mg 100–150 mg 30–50 mg 150 mg 20 mg 100–200 mg 30 mg 300–600 mg 20–30 mg	Depressive patients (n = 44)	44 (mean age)	Other	PP	Long-term treatment (n. spec.) = 18% of patients showed severe impairment No differences between treatment groups (TCAs, SSRIs, MAOI)
Brunnauer and Laux (2003)	Amitriptyline Doxepin Citalopram Fluoxetine Fluvoxamine Paroxetine Mirtazapine Venlafaxine + comedication	60–180 mg 75–225 mg 20–40 mg 20–60 mg 150–300 mg 20–40 mg 20–60 mg 150–375 mg	Depressive patients (n = 64)	25–77	Other	PP	Long-term treatment (n. spec.) = 20% of patients showed severe impairment TCAs > selective antidepressants
Miyata et al. (2018)	Antidepressants + comedication	n. spec.	Depressive patients (n = 65)	24–57	Other	DS	Long-term treatment n. spec.) = not inferior to healthy controls with respect to driving performance
van der Sluiszen et al. (2020)	Amitriptyline Mirtazapine + comedication Amitriptyline Mirtazapine + comedication	Treatment < 3 y 32.5 ± 29.0 22.0 ± 12 Treatment > 3 y 63.3 ± 52.7 26.7 ± 12.5	Patients (n = 38)	54,0 (mean age)	Other		Long-term treatment (n. spec.) = no impairment in all antidepressant users Long-term treatment (n. spec.) = impairment when treated <3 y
Mood–stabilizer.							
Comparative clinical studies							
Hatcher et al. (1990)	Lithium	400–1250 mg	Bipolar (n = 16)	18–63	Other	DS	Long-term treatment (n. spec.) = impairment
Jauhar et al. (1993)	Lithium	Serum level 0.5–1.5 mmol/L	Bipolar and unipolar (n = 20)	45 (mean age)	Other	DS	Long-term treatment (n. spec.) = impairment
Segmiller et al. (2013)	Lithium Lamotrigine	600–1000 mg 200–400 mg	Bipolar (n = 24)	25–63	Other	PP	Long-term treatment (n. spec.) = 17% of patients severely impaired Better test performance of patients treated with lamotrigine
Benzodiazepines							
Tranquilizer							
van Laar et al. (1992)	Diazepam	15 mg	Generalized anxiety disorder (n = 12)	18–50	DBC	ORT	Acute treatment (d) = impairment Subchronic treatment (d) = impairment Long-term treatment (d) = no impairment
Moore (1977)	Medazepam	5–30 mg	Anxiety patients (n = 14)	20–40	DBC	SORT DS	Subchronic treatment (d) = impairment Subchronic treatment (d) = no impairment
Hypnotics							
Schmidt et al. (1986)	Flunitrazepam	2 mg	Insomniacs (n = 16)	n. spec.	RDB	ORT	Acute treatment (n) = impairment
Vermeeren et al. (1995)	Flunitrazepam	2 mg	Insomniacs (n = 17)	25–51	DBC	ORT	Acute treatment (n) = no impairment
Brookhuis et al. (1990)	Flurazepam	30 mg	Insomniacs (n = 16)	26–41	DBC	ORT	Acute treatment (n) = impairment
Brookhuis et al. (1990)	Lormetazepam	1/2 mg	Insomniacs (n = 32)	26–41	DBC	ORT	Acute treatment (n) = no impairment (1 mg) Acute treatment (n) = impairment (2 mg) no residual effect was found in the afternoon
Staner et al. (2005)	Lormetazepam	1 mg	Insomniacs (n = 23)	38.8 (mean age)	RDBC	DS	Acute treatment (n) = impairment
Partinen et al. (2003)	Temazepam	20 mg	Insomniacs (n = 18)	35–58	RDBC	DS	Acute treatment (n) = no impairment
Schmidt et al. (1986)	Temazepam	20 mg	Insomniacs (n = 16)	n. spec.	RDB	ORT	Acute treatment (n) = no impairment
**Z–drugs**							
Partinen et al. (2003)	Zolpidem	10 mg	Insomniacs (n = 18)	35–58	RDBC	DS	Acute treatment (n) = no impairment
Staner et al. (2005)	Zolpidem	10 mg	Insomniacs (n = 23)	38.8 (mean age)	RDBC	DS	Acute treatment (n) = no impairment
Vermeeren et al. (1995)	Zolpidem	10 mg	Insomniacs (n = 17)	25–51	DBC	ORT	Acute treatment (n) = no impairment
Leufkens et al. (2014a)	Zopiclone	7,5 mg	Insomniacs (n = 32)	52–71	DBC	ORT	Acute treatment (n) = impairment Magnitude of impairment significantly less in chronic hypnotic users group
Staner et al. (2005)	Zopiclone	7.5 mg	Insomniacs (n = 23)	38.8 (mean age)	RDBC	DS	Acute treatment (n) = impairment
Comparative clinical studies (hypnotics)							
Leufkens et al. (2014b)	Zopiclone Temazepam Midazolam Oxazepam Zolpidem Lormetazepam Loprazolam Clonazepam Flurazepam Nitrazepam	n. spec.	Insomniacs (n = 42)	50–75	Other	ORT	Long-term treatment (n. spec.) = no difference between frequent or infrequent users and healthy controls
Treatment combinations (BZDs/Z-drugs and comedication)							
van der Sluiszen et al. (2019)	Alprazolam Bromazepam Brotizolam Diazepam Lorazepam Lormetazepam Midazolam Nitrazepam Oxazepam Temazepam Zolpidem Zopiclone + comedication	n. spec.	Patients (n = 44)	Hypnotic group: 55.6 y (mean age) Anxiolytic group: 55.2 y (mean age)	Other	ORT	Long-term treatment (n. spec.) = impairment with hypnotics (treatment <3 y and >6 mo) Long-term treatment (n. spec.) = no impairment with hypnotics (treatment >3 y) Long-term treatment (n. spec.) = no impairment with anxiolytics

Abbreviations: Aps, antipsychotics; BZDs, benzodiazepines; CPÄ, chlorpromazine-equivalents; DBC, double-blind crossover; DS, driving simulator; d, during the day (i.e., dosages were given at the day of driving assessment); FGAs, first-generation antipsychotics; n, nocturnal (i.e., dosages were given in the night before driving assessment); n. spec., not specified; ORT, on-road test; Other, cross-sectional or open-label study; PP, psychomotor performance; RCT, randomized clinical trial; RDB, randomized double blind; RDBC, randomized double-blind crossover; SGAs, second-generation antipsychotics; SORT, simulated on-road test.

^*a*^Outcome: acute mean day 1–7; subchronic mean day 8–21; long-term mean >21 days.

## Antipsychotics

### Comparative Clinical Studies

Psychomotor functions according to legal regulations or driving simulator performance under long-term treatment with antipsychotic monotherapy were solely investigated within a naturalistic, non-randomized design, and patients were not investigated before and after treatment with respect to driving performance.

Under long-term treatment with antipsychotics and stabilized psychopathological conditions, schizophrenic and schizoaffective patients showed severe impairments in psychomotor functions related to driving skills in 31% (27%–42.5%) of cases. Second-generation antipsychotics (SGAs) seemed to be advantageous compared with first-generation antipsychotics (FGAs). Twenty-three percent (15%–27%) of patients treated with SGAs compared with 42% (34%–60%) of patients treated with FGAs showed severe impairments (Brunnauer et al., 2004, 2005, 2009, 2012). Differences could also be seen on driving simulator performance, with better results in patients treated with SGAs compared with FGAs in a risk simulation (Brunnauer et al., 2005, 2009). Although in 1 study an advantage of clozapine on reactivity within SGAs could be shown (Brunnauer et al., 2004), altogether, no relevant differences with respect to driving performance between SGAs investigated was evident (Brunnauer et al., 2009; Brunnauer and Laux, 2012).

### Treatment Combinations

While polydrug treatment is frequent in clinical practice, only a few studies could be identified that investigated driving performance under common psychopharmacologic treatment combinations in schizophrenic or schizoaffective patients. Irrespective of drug regimen, most patients do not seem to reach the level of psychomotor or driving simulator performance of healthy controls following subchronic or long-term polydrug treatment and psychopathologic stabilization (Hobi et al., 1981; Grübel-Mathyl, 1987; Wylie et al., 1993; Soyka et al., 2005; Fuermaier et al., 2019). In clinical routine settings, where patients were co-medicated with lithium, valproate, hypnotics, or antidepressants in addition to treatment with SGAs or FGAs, 32%–54% of patients showed a very low test performance in functional domains relevant for driving, with a better performance of patients under polydrug treatment with SGAs (Grabe et al., 1999; Kagerer et al., 2003).

## Antidepressants

### Trizyclic Antidepressants (TCAs)/Amitriptyline

The acute and subchronic effects of low-dose nocturnally administered amitriptyline on driving performance have been investigated in chronic pain patients. Amitriptyline acutely impaired performance in an on-road driving test that diminished after subchronic treatment. It is important to note that patients’ pain intensity ratings were unexpectedly low and moreover did not diminish under amitriptyline treatment; thus, there might have been a selection bias with respect to participants (Veldhuijzen et al., 2006).

### Agomelatine

A novel approach to treat depression, focusing on circadian rhythms by melatonergic mechanisms, has been the development of agomelatine. After 4 weeks of treatment, patients significantly improved in both symptomatology and psychomotor skills relevant for driving, with most benefits occurring within the first 2 weeks of treatment. A functional level in psychomotor performance comparable with healthy controls could not be reached at the end of the 4-week study phase. In the on-road driving test in real traffic, patients were not significantly inferior to healthy controls and could in most cases be rated by a licensed driving instructor as sufficiently fit to drive after 4 weeks of treatment (Brunnauer et al., 2015).

### Mirtazapine

Psychomotor and driving simulator performance in depressive patients during 14 days of treatment significantly improved under the noradrenergic and specific serotonergic antidepressant mirtazapine, indicating that partly remitted depressed patients show a better driving performance compared with untreated patients. Performance level in psychomotor functions of healthy controls was not reached within the subchronic or long-term treatment phase (Brunnauer et al., 2008). However, after 2 weeks of treatment, differences compared with healthy controls were not evident in driving simulator performance (Brunnauer et al., 2008; Shen et al., 2009).

### Reboxetine

The effects of the selective noradrenergic reuptake inhibitor reboxetine on psychomotor function and driving simulator performance were investigated in a randomized comparative clinical study in patients with major depression. After 14 days of treatment, patients improved in driving skills, especially in tests measuring selective attention and reactivity. Furthermore, the frequency of accidents in the risk simulations with a driving simulator markedly decreased. Although patients were still inferior to healthy control subjects in psychomotor performance after 14 days of treatment, no differences could be shown in the driving-simulator tasks (Brunnauer et al., 2008).

### Trazodone

Patients with primary insomnia treated with nocturnally administered trazodone performed slightly worse in a short-term memory task, verbal learning, body sway, and arm muscle endurance. Negative effects on driving simulator performance were not found within 7 days of treatment. There was, however, a high dropout rate; 47 out of 63 individuals did not complete the entire study (Roth et al., 2011).

### Venlafaxine

Four weeks of treatment with the serotonin norepinephrine reuptake inhibitor venlafaxine had positive effects on depressive symptoms and driving skills; most improvements were observable in the first 2 weeks. Although patients did not achieve healthy controls performance in psychomotor tasks within 4 weeks of treatment, significant differences could not be seen in an on-road test in real traffic compared with healthy participants’ performance (Brunnauer et al., 2015).

### Comparative Clinical Studies

The effects of long-term treatment with antidepressant monotherapy on psychomotor function in a clinical routine setting were investigated by Brunnauer et al. (2006). Sixteen percent of inpatients under pharmacologic steady-state conditions and prior to discharge showed severe impairments and thus had to be estimated as “unfit to drive.” Controlling for confounding factors, a total of 28% of patients passed psychomotor performance tests without major impairments; 10% showed no major impairments in the TCA-group and 37.5% in the modern antidepressant group.

Performance in on-road tests was assessed in 2 studies indicating that patients treated with selective serotonin reuptake inhibitors (SSRIs) or the serotonin norepinephrine reuptake inhibitor venlafaxine were inferior to healthy controls. Differences between treatment groups could not be shown, and driving impairment was significantly less in the treatment group compared with untreated depressive patients (Wingen et al., 2006; van der Sluiszen et al., 2017b).

### Treatment Combinations

Grabe et al. (1998) did not identify differences between patients treated with TCAs, SSRIs, monoamine oxidase inhibitors, and common co-medications, whereas Brunnauer and Laux (2003) have shown less psychomotor performance impairment in patients treated with SSRIs and co-medication—approximately 37% were without major impairment—compared with treatment with TCAs (12%). No differences compared with healthy control patients could be shown in driving simulator performance (Hobi et al., 1981, Miyata et al., 2018) or in an on-road tests with patients treated long term (>3 years) with sedating antidepressants combined with TCAs, SGAs, or modern antidepressants (van der Sluiszen et al., 2020).

The combined use of fluoxetine or moclobemide and benzodiazepines was differentially assessed in an on-road test by Ramaekers et al. (1997). In this study, 79% patients under fluoxetine and 73% under moclobemide additionally used benzodiazepines during treatment. Post-hoc analysis revealed that besides a linear decrease in driving performance throughout the treatment period in both groups, there was an apparent difference in patients taking competitive benzodiazepine co-medication metabolized by a P450 isozyme subject to inhibition by the antidepressant. Patients treated with fluoxetine decreased in driving performance until week 3 of treatment that diminished at week 6; under fluoxetine and competitive benzodiazepine treatment, a decline throughout the 6-week treatment period could be seen.

## Mood Stabilizers

### Comparative Clinical Studies

Seventeen percent of euthymic bipolar patients under long-term treatment with lamotrigine or lithium were severely impaired in psychomotor function relevant for driving, with a considerable advantage for patients treated with lamotrigine (Segmiller et al., 2013). Significant impairments compared with healthy controls could also be shown in driving simulator performance of bipolar and unipolar patients on stable lithium therapy (Hatcher et al., 1990; Jauhar et al., 1993).

## Benzodiazepines and Z-Drugs

### Tranquilizers

#### Diazepam

Outpatients suffering from anxiety disorders showed a significant impairment in the first 3 weeks of treatment in an on-road driving test that diminished after week 3 (van Laar et al., 1992).

#### Medazepam

Subchronic treatment with medazepam in anxious patients showed that driving simulator performance was not adversely affected, while in a simulated on-road test, a minor increase of errors was discerned. As the author states, mean dosages were at a very low therapeutic level and thus general conclusions with respect to treatment effects should be drawn cautiously (Moore, 1977).

### Hypnotics

#### Flunitrazepam

Patients with insomnia experienced a slight impairment of driving performance in an on-road test when treated for 7 days with flunitrazepam (Schmidt et al., 1986). On the contrary, a single dose administered on retiring to bed to patients with insomnia did not significantly affect driving performance the next morning (Vermeeren et al., 1995).

#### Flurazepam

Flurazepam had acute effects on driving performance in patients with insomnia. The ability to control the lateral position of the vehicle was significantly impaired, and the degree of impairment was more pronounced in females. Moreover, impairment was greater in the morning (10 to 11 hours) than in the afternoon (16 to 17 hours) after administration (Brookhuis et al., 1990).

#### Lormetazepam

Acute treatment with lormetazepam 1 mg did not impair driving performance in an on-road test of patients with insomnia when tested 10 to 11 hours and 15 to 16 hours post administration. However, 7 days of bedtime administration of 1 mg lormetazepam in primary insomnia patients induced significant impairment in driving simulator performance (Staner et al., 2005). Acute treatment with 2 mg impaired driving performance of insomniacs 10 to 11 hours post ingestion, but no residual effects after 15 to 16 hours were evident (Brookhuis et al., 1990).

#### Temazepam

There were no significant residual effects on performance in a driving simulation test 5.5 hours after intake of temazepam the next morning (Partinen et al., 2003), and no decrease of driving performance during an on-road test was found in patients with sleep disorder that received temazepam once a night for 7 days (Schmidt et al., 1986).

#### Zolpidem

Single-dose administration as well as repeated (7 days) treatment with zolpidem given at nighttime did not impair on-road or driving simulator performance of insomnia patients (Vermeeren et al. 1995; Partinen et al., 2003; Staner et al., 2005).

#### Zopiclone

A single, oral overnight dose of zopiclone significantly impaired on-road driving performance in insomnia patients who chronically used hypnotics as well as in patients who did not or only infrequently used hypnotics. However, the magnitude of impairment was less in the chronic users group (Leufkens et al., 2014a). Analogous results were obtained by Staner et al. (2005), who showed that single bedtime administration of zopiclone induced next-day driving impairments on a driving simulator.

### Comparative Clinical Studies

Actual driving performance and driving-related skills of elderly chronic hypnotic users were investigated by Leufkens et al. (2014b). Patients were divided in frequent users (using hypnotics ≥4 nights/wk) and infrequent users (using hypnotics ≤3 nights/wk). Driving, measured by a standardized highway driving and a car-following test, was not impaired in patients with insomnia irrespective of the frequency of use of hypnotics.

### Treatment Combinations

Van der Sluiszen et al. (2019) suggested that impairment of driving performance was dependent on treatment duration in elderly patients. While the ability to drive was significantly impaired in patients treated with hypnotics and comedication for less than 3 years, no deterioration was found in patients treated for more than 3 years. In long-term users (>6 months) of anxiolytics and comedication, no clinically relevant differences in driving performance were evident compared with healthy controls.

Table 2 gives an overview of controlled experimental patient studies on antipsychotics, antidepressants, mood stabilizers, benzodiazepines, Z-drugs, and driving performance.

**Table 2.** Summary of Results of Controlled Experimental Patient Studies on Monotherapy with Antipsychotics, Antidepressants, Mood–Stabilizers, Benzodiazepines and Z–Drugs on Driving Performance

Substance	No. of investigations	Acute effects^*a*^	Subchronic–/ long– term–effects^*a*^	Checked doses (mg)	Therapeutic range (mg)
Antipsychotics: no data available					
Antidepressants					
Amitriptyline	1	↓	↔	25	50–225
Agomelatine	1	––––	↑	25–50	25–50
Mirtazapine	2	↑	↑	30–60	15–45
Reboxetine	1	–––	↑	2–8	4–10
Trazodone	1	↔ (high dropout rate)	–––	50	150–600
Venlafaxine	1	–––	↑	150–300	75–375
MAO–inhibitors: no data available					
Mood stabilizers: no data available					
Benzodiazepines – tranquilizers					
Diazepam	1	↓	↓(until week 3)	15	5–20
			↔ (after week 3)		
Medazepam	1	–––	()	5–30	10–30
Benzodiazepines – hypnotics					
Flunitrazepam	2	()	–––	2	0,5–1
Flurazepam	1	↓	–––	30	15–30
Lormetazepam	2	1 mg ()	–––	1/2	0,5–2
		2 mg ↓			
Temazepam	2	↔	–––	20	10–40
Z–drugs					
Zolpidem	3	↔	–––	10	5–10
Zopiclone	2	↓	–––	7,5	3,75–7,5

Abbreviations: –––, no data available; (), inconsistent data; ↔, no impairment; ↓, impairment; ↑, improvement.

^*a*^Treatment effects: acute mean day 1–7, subchronic mean day 8–21, long-term mean >21 days.

## Discussion

### Summary of Findings

Only 40 studies could be found according to our selection criteria, indicating a clear lack of patient studies on driving performance under psychopharmacologic treatment. No data were available that give information about causal relationships between antipsychotics, monoamine oxidase inhibitors, mood stabilizers, and driving performance.

#### Antipsychotics

Overall, with regard to antipsychotic medication, a great proportion of schizophrenic and schizoaffective patients does not reach the level of psychomotor or driving simulator performance of healthy controls following long-term treatment despite significant clinical improvement (Wylie et al., 1993; Soyka et al., 2005; Fuermaier et al., 2019). Keeping in mind the great heterogeneity between studies, on average 31% (27%–42.5%) of in-patients showed severe impairment of psychomotor function relevant for driving under steady-state pharmacological conditions prior to hospital discharge; that is, patients were treated in most cases 6 weeks and more in a stationary setting (Brunnauer et al., 2004, 2005, 2009, 2012; Soyka et al., 2005). Considering that approximately 32% of young, unmedicated schizophrenic patients showed severe impairment of psychomotor skills relevant for driving (Segmiller et al., 2017), it can be assumed that impairments under steady-state pharmacologic conditions may be primarily due to the illness itself rather than to treatment effects.

Under common treatment combinations, a greater proportion of patients (32%–54%) showed severe impairment in functional domains relevant for driving (Grabe et al., 1999; Kagerer et al., 2003). As a polypharmacological approach is often administered in difficult-to-treat patients with poor treatment response, a selection bias towards a more severe clinical course might be discussed.

It is still a matter of debate whether SGAs have an advantage with respect to psychomotor function and cognition compared with FGAs (e.g., Keefe et al., 2007). In this review, better driving performance was related to treatment with SGAs (amisulpride, clozapine, olanzapine, quetiapine, risperidone, sertindole, zotepine) compared with FGAs (haloperidol, flupenthixol, zuclopentixol) (Brunnauer et al., 2004, 2005, 2009, 2012; Soyka et al., 2001, 2005). Under polydrug treatment, an advantage for SGAs was also evident (Grabe et al., 1999; Kagerer et al., 2003). Although there was a slight advantage of patients treated with clozapine in psychomotor skills relevant for driving in 1 study (Brunnauer et al., 2004), altogether, differences between SGAs with respect to driving performance could not be shown (Brunnauer et al., 2009; Brunnauer and Laux, 2012).

#### Summary

On average, one-third of schizophrenic or schizoaffective patients under treatment with antipsychotics showed severe impairment in skills relevant for driving compared with healthy controls, which may be primarily due to residual symptoms of the illness itself rather than to negative side effects of antipsychotic treatment. Studies point to an advantage of SGAs compared with FGAs with respect to driving skills, and there seems to be a disadvantage under antipsychotic polypharmacy. However, conclusions must be drawn cautiously with regard to the marked methodologic heterogeneity of the studies. Moreover, interpretation of data is also limited since adverse effects of pharmacological treatment and illness-associated impairment cannot be disentangled within studies under review.

### Antidepressants and Mood-Stabilizers

Approximately 16% of patients with major depression and 17% of bipolar patients showed severe impairment in psychomotor skills relevant for driving under long-term monotherapy with antidepressants or mood-stabilizers (Brunnauer et al., 2006; Segmiller et al., 2013). Data point to an advantage of treatment with modern antidepressants and SSRIs in particular over TCAs with regard to driving skills (Brunnauer and Laux, 2003; Brunnauer et al., 2006). Although Ramaekers et al. (1997) showed that specific combinations of antidepressants with benzodiazepines may have deleterious effects on driving performance, there is also evidence that under treatment combinations with lithium, carbamazepine, neuroleptics, and hypnotics—when given on clinical considerations—a similar distribution of patients showed severe impairment (18%–20%) compared with patients under antidepressive monotherapy (Grabe et al., 1998; Brunnauer and Laux, 2003).

Acute treatment with amitriptyline, but not subchronic treatment, had negative effects on the on-road performance of insomnia patients (Veldhuijzen et al., 2006). Under trazodone, no impairment in driving simulator performance could be seen in the acute treatment phase (Roth et al., 2011).

Patients significantly improved in psychomotor functions and driving simulator performance under subchronic or long-term treatment with agomelatine, mirtazapine, reboxetine, or venlafaxine (Brunnauer et al., 2008, 2015; Shen et al., 2009). Results with respect to achievement of functional level of healthy controls in driving-simulator or on-road performance are mixed, showing either lower performance (Wingen et al., 2006; van der Sluiszen et al., 2017b) or no significant differences (Hobi et al., 1981; Brunnauer et al., 2008, 2015; Miyata et al., 2018; van der Sluiszen et al., 2020).

No patient data were available that give information about causal relationships of SSRI monotherapy on driving ability in patients.

Under long-term treatment with lithium, unipolar and bipolar patients showed a significantly lower performance than matched healthy volunteers in driving simulator performance, and there seems to be an advantage when patients were treated with lamotrigine compared with lithium (Hatcher et al., 1990; Jauhar et al., 1993; Segmiller et al., 2013).

#### Summary

Patients definitively profit within 2–4 weeks from treatment with antidepressants with respect to driving performance, and data point to an advantage when treated with modern antidepressants over TCAs and with lamotrigine over lithium. Although level of psychomotor performance of healthy controls was in at least a subgroup of (partly) remitted patients not reached, differences in driving simulator performance or on-road tests were less pronounced or even did not exist and thus may be an indication for compensational competencies with respect to driving performance in a “real world context.” Not least, there is also evidence that the effects of sedating antidepressants on driving performance attenuate over time. Co-administration of other psychotropic medicines with antidepressants, when given on clinical considerations, does not seem inferior compared with trials on monotherapy.

## Benzodiazepines and Z-Drugs

### Tranquilizers

Elderly patients treated long term (>6 months) under clinical conditions, that is, most were comedicated with antidepressants or antipsychotics, did not differ in driving performance compared with age-matched healthy controls (van der Sluiszen et al., 2019). Results of controlled experimental studies suggest that subchronic or long-term treatment with low-dose medazepam did not impair on-road driving performance of anxious patients (Moore, 1977). However, deleterious effects on driving performance in an on-road test could be shown the first 3 weeks of treatment with diazepam (van Laar et al., 1992).

### Hypnotics

Most studies investigated effects of hypnotics either in a single-dose regimen or after 7 days of treatment in patients with insomnia. Temazepam and zolpidem seem to be free of acute negative effects on driving performance when given to patients with a sleep disorder (Vermeeren et al., 1995; Partinen et al., 2003; Staner et al., 2005). Zopiclone, flurazepam, and dose-dependent lormetazepam cause significant driving impairment in the acute treatment phase of patients with insomnia (Brookhuis et al., 1990; Staner et al., 2005; Leufkens et al., 2014a). When given as a single nocturnal dose, flunitrazepam seems to be free of deleterious effects on driving performance the next morning (Vermeeren et al., 1995); this does not appear to be true, however, when treated for 7 consecutive days (Schmidt et al., 1986).

There is some evidence that frequency of hypnotic intake does not play a major role in driving performance in elderly patients (Leufkens et al., 2014b) and that in chronic elderly users (>3 years), no driving impairments seem to become evident (van der Sluiszen et al., 2019). This is in line with studies indicating development of tolerance over time in long-term users (Pomara et al., 2015; van der Sluiszen et al., 2017a). However, there is also evidence that tolerance with respect to skills relevant for driving may be only partial (van der Sluiszen et al., 2017a).

#### Summary

Diazepam significantly worsened driving the first 3 weeks of treatment, whereas low-dose medazepam did not impair driving. Among hypnotics, flunitrazepam, flurazepam, lormetazepam (dose dependent), and zopiclone significantly impaired driving performance at treatment initiation, whereas temazepam and zolpidem were free of deleterious effects in patients with insomnia. There is evidence in long-term users that impairing effects on driving attenuate over time, which may be only partial.

### Limitations

Major limitations of the current analysis are the small database and methodological constraints–60% of studies were cross-sectional or open-label trials—that do not allow to disentangle treatment effects from illness-associated impairment. Included studies show large variations with study designs, patient characteristics, and methodology used to measure driving performance. Besides, poor description of patient samples, with respect to clinical data (e.g., psychopathologic symptoms at time of testing, duration of illness, or driving characteristics) are missing in most cases. Thus, because of heterogeneity of studies, the summarized evidence was not suitable for a meta-analytic approach. It should also be noted that the literature review was restricted to PubMed database. Albeit screening of reference lists of included studies and related reviews did not lead to an inclusion of further studies in our review, it could be argued that the utilization of other databases might have provided additional information. Considering all these shortcomings, conclusions of this review should be drawn cautiously.

## Conclusion

Much more controlled patient studies are needed to analyze the complex relationship between illness and medication to clarify a core issue: from which pharmacologic treatment do patients benefit, also with respect to driving performance. The current synopsis gives evidence that psychopharmacologic medicines under review improve or at least stabilize driving performance of patients when treated long-term under clinical considerations. To enhance treatment compliance and give information to professionals, patient information leaflets as well as classification systems of potential driving impairing medicines should also incorporate information about the stabilizing effects of long-term treatment on driving performance and recommendations on the passage of time that should be considered to minimize possible detrimental effects.

Due to the fact that particular drugs can place patients at risk—at least after treatment initiation—during everyday activities like driving a car, it is of crucial importance to ensure that physicians are aware of this and that they try to minimize potential disruptive effects on neuropsychological processes. Prescribers of drugs should be recommended to minimize the number of prescribed drugs, keeping in mind potential pharmacokinetic interactions, to adjust dosage regimens according to a patient’s individual response, and to administer medicines in nocturnal doses whenever possible—for example, in the case of sedating antidepressants—to mitigate adverse side effects on driving performance. Not least, patients should be educated on the potential risks of psychopharmacological treatments with respect to driving to attain a better awareness and self-monitoring of possible adverse drug effects.

## Supplementary Material

pyab031_suppl_Supplementary_Material

## Statement of Interest

During the last 3 years, A.B., F.H., and G.L. have no conflicts of interest to declare for this study. P.Z. has received speaker fees or honoraria for advisory board participation over the last 3 years from Janssen Pharmaceuticals, Schwabe, Servier, and Neuraxpharm. All these affiliations have no relevance to the work covered in the manuscript.
