# Supplementary material for: Driving Performance Under Treatment of Most Frequently Prescribed Drugs for Mental Disorders: A Systematic Review of Patient Studies
Source: Int J Neuropsychopharmacol. 2021 May 26;24(9):679–93. doi: 10.1093/ijnp/pyab031 (PMC8453274; doi:10.1093/ijnp/pyab031)
Supplement: pyab031_suppl_Supplementary_Material [file pyab031_suppl_supplementary_material.docx]

**Supplementary Material**

**Additional search terms used for identifying articles:**

“driving performance”, “driving ability”, “driving skills”, “fitness to drive”, “traffic safety”, “on the road driving test”, “driving simulation” AND

Agomelatine, amitriptyline, bupropion, carbamazepine, citalopram, clomipramine, dothiepin, duloxetine, escitalopram, esketamine, fluoxetine, fluvoxamine, imipramine, lamotrigine, lithium, lofepramine, maprotilin, mianserin, milnacipran, mirtazapine, moclobemide, nortriptyline, paroxetine, rasagiline, reboxetine, selegeline, sertraline, sulpirid, tranylcypromine, trazodone, trimipramine, valproate, venlafaxine, vortioxetine,

Amisulpride, aripiprazole, asenapine, benperidol, brexpiprazole, bromperidol, cariprazine, chlorpromazine, clozapine, droperidol, flupentixol, fluphenazine, fluspirilene, haloperidol, levomepromazin, loxapine, lurasidone, melperone, olanzapine, paliperidon, perazine, perphenazine, pimozide, pipamperone, promethazine, prothipendyl, quetiapine, risperidone, sertindole, sulpiride, thioridazine, thiothixene, tiapride, ziprasidone, zotepine, zuclopenthixol,

Alprazolam, bromazepam, brotizolam, camazepam, chlordiazepoxide, clobazam, clonazepam, clorazepat, delorazepam, diazepam, dikaliumclorazepat, estazolam, fludiazepam, flunitrazepam, flurazepam, halazepam, ketazolam, loprazolam, lorazepam, lormetazepam, medazepam, mexazolam, midazolam, nitrazepam, oxazepam, prazepam, temazepam, tetrazepam, triazolam, zaleplon, zolpidem, zopiclone.
